# Editorial

**Published:** 1899-01

**Authors:** 


### Editorial.

Dr. William Osler has been appointed Dean of the Johns Hopkins University Medical Department, to succeed Dr. W. H. Welch.

The Antikamnia Company are sending their friends their skeleton calendar for 1899. It a gruesome if artistic reminder of the transitoriness of things temporal.

Dr. Henry C. Doughty, of Augusta, died recently from tuberculosis inoculated in the process of his work in the pathological laboratory of the Augusta Medical College. Another martyr to science.

The "The Physician's Daily Memorandum" is the title of a convenient little book sent by the M. J. Breitenbach Company. It is a handy thing to have on the doctor's desk and serves to remind him of the value of Pepto-mangan.

We have received a letter from Dr. Tircuit, of Oubre, Louisiana, enclosing $1.00 for subscription for 1899. Dr. Tircuit has been a subscriber for nineteen years and has always paid in advance. If all of our subscribers followed his example, what an almighty cinch medical journalism would be!

Dr. John B. Hamilton died at Elgin, Ill., Dec. 24, 1898. He was for a long time supervising surgeon of the Marine Hospital Service. He was also Professor of Surgery in Rush Medical College, Superintendent of the Insane Asylum at Elgin, Ill., and editor of the *Journal of the American Medical Association*. His loss will be regretted by the whole profession.

The recent election of officers of the Atlanta Medical Society resulted as follows:

President—Dr. W. S. Goldsmith.
Vice-President—Dr. Katherine Collins.
Secretary—Dr. Claude A. Smith.
Treasurer—Dr. E. Van Goidtsnoven.
Librarian—Dr. Michael Hoke.

---

### ANNOUNCEMENT.

We are pleased to announce that the business management of this journal will be hereafter entrusted to Dr. Michael Hoke. Dr. Hoke is a graduate of the University of Virginia and served for two years on both the surgical and medical sides of the Johns Hopkins Hospital, and is at present lecturer on Pathology in the Atlanta College of Physicians and Surgeons. Dr. Hoke has always been interested in journalistic work, and in the future will not only conduct the business affairs of the RECORD, but will lend his valuable assistance in the editorial and literary department as well. After four years of steady diet served out by the present incumbent to subscribers, they will be pardoned for making this addition the subject of Shakespeare's remark, "For this relief much thanks."

---

Our esteemed contemporary the *Atlanta Medical and Surgical Journal* announces itself as "the official organ of the State Board of Medical Examiners." We ask, in a spirit of inquiry, what beyond publishing questions given on the examination which are common property and can serve no purpose beyond offering something of a guide to prospective examinees, are the duties of the official organ?

As applicants for examination for license to practice in this State are usually lately hatched doctors whose sense of obligation to their congeners has not been developed to the point of subscribing for a medical journal conducted by the profession and for the profession, it seems to us that our e. c. might fill its *la cunæ* with matter of more interest to its patrons.

It was precisely for these reasons that we recently declined to be the mouthpiece of the Society for the Distribution of Pajamas among the Hot Water Cannibals.

### "INTERFERENCE" OR "INTERVENTION."

The editor of the *Richmond Journal of Practice* finds fault with the word *interference* as applied to surgery and proposes *intervention* as more aptly expressing the act. He quotes Webster and Trench in support, though to us the quotations from these sources, referring to the act in the abstract rather than the concrete, fail to fortify his position. Interference by word conveys a different shade of meaning from interference by deed. Etymologically, interference (from inter and fero) in the mechanical sense is to be preferred to intervention (from inter and venio). The one is derived from a transitive verb and bears with it the sense of material and actual aid; the other from an intransitive source and expresses potentiality. I carry (something) between, and I come between. Of the two words, to our mind interference is the stronger and more nearly expresses the act.

But the Richmond editor has caught up only a ripple cast shoreward from the vast sea of inconsistencies in medical phraseology. Without pausing to consider what we are saying, we daily use words culled from every source and all times in the progress of medicine, and which are often the relics of long-since exploded theories and just as often mere nonsense. There is nothing sacred about the sacrum except its name, which, considering its location in the body, is almost a blasphemous joke, nor has aponeurosis anything whatever to do with a nerve. The tendo achillis comes from the mythical story of the death of Achilles from a wound of the heel, his only vulnerable part; this secret having been divulged to his enemies by a woodpecker! What has hysteria to do with the womb, or lunacy with the moon? Gonorrhoea is not a seminal discharge; the surgeon as well as every other man "works with the hands," and the rectum is not straight. We speak of an action meaning a fecal evacuation while manifestly the "stool" is the result of action, not the action itself. Except as it steals our rest, what has a furuncle to do with a little thief, and what is the connection between chondrology and a grain of wheat?

A word should have some real connection with the thing designated. How far do the words quoted carry out this requirement? The symbol ℞ placed at the head of a prescription is usually regarded as an abbreviated form of recipe, but is in fact a sort of obsolete talisman. The original form was the letter Z, the initial of Zeus and the symbol of the planet Jupiter (Sozinsky), which was believed to have a favorable influence over disease. Even the emblematic Esculapian serpent is a nasty fraud, as it is derived from the representations of the phallus. Our generic word fornication is derived from fornix, an arch, from the custom of the Roman prostitutes of the lower class enticing men to partake of their wretched "favors" in dark crypts and squalid archways. We use the term Cæsarean section from the hazy tradition of Cæsar having been "from his mother's womb untimely ripped."

The average medical prescription is a grand polyglot *pot-pourri*, the case endings of the Latin varying from nominative to ablative according as the fancy of the writer dictates, the breaches of ignorance of the ancient tongue being deftly filled with safe and sufficient English. The Roman and Arabic numerals foregather peacefully and all go to make one harmonious whole—one "dost." Why should the druggist be selected for this anachronistic infliction? Why not address our orders to the grocer and the butcher in Anglo-Latin? It would be just as consistent to send to the butcher an order for two pounds of tripe writing ℞ intestini preparati lb. ij as to the druggist on election day for Spt. Frumenti ℥viii.

Without prolonging this painful subject, would it not be better in cleaning out the stables of Augeus, to begin at the piles of compost rather than picking up the scattered straws?

